# Rewarding recovery: the time is now for contingency management for opioid use disorder

**DOI:** 10.1080/07853890.2022.2068805

**Published:** 2022-04-26

**Authors:** Steven L. Proctor

**Affiliations:** a Thriving Mind South Florida, Miami, FL, USA; b PRO Health Group, Miami Beach, FL, USA; c Department of Psychiatry and Behavioral Health, Florida International University, Herbert Wertheim College of Medicine, Miami, FL, USA

**Keywords:** Contingency management, overdose crisis, opioid use disorder, recovery, rewards, technology, innovation

## Abstract

Contingency management (i.e. rewarding people, often with money, for achieving their recovery goals) is backed by decades of empirical support yet remains highly underutilized. Rewards are rarely used in real-world clinical practice due to a number of concerns, including most notably, the apparent lack of innovation, as well as moral, philosophical, ethical, and economic concerns, and even federal rules meant to prevent illegal inducements in health care. Still, other opponents argue that some patients will try to "game" the system by simply doing whatever it takes to earn monetary rewards. This paper provides a succinct, up-to-date overview of the current evidence base for contingency management for opioid use disorder. Common barriers and solutions to implementation, as well as implications for future research and clinical practice are discussed. Although important, greater uptake of contingency management interventions is about more than legislation and regulations; it’s about recognizing stigma, shaping attitudes, and increasing awareness. Provider involvement in advocacy efforts at all levels and collaboration involving academic–industry partnerships is necessary to advance the burgeoning digital health care space and improve outcomes for people with opioid use disorder.

**Key Messages**
- Contingency management is highly effective but highly underutilized.
- Low uptake is largely attributed to a lack of innovation and moral, ethical, and economic concerns, among other barriers.
- Technology-enabled solutions and academic–industry partnerships are critical to advance opioid use disorder care.

America’s drug overdose crisis—largely driven by opioids—is arguably one of the greatest public health concerns of our time with significant loss of life and economic burden. Over100,000 Americans died of a drug overdose in the past year with roughly 75% attributed to opioids [1], and the estimated annual opioid-related cost to U.S. society is upwards of $1.02 trillion [2,3]. America’s response to the escalating opioid overdose crisis has reached a tipping point. At such a critical juncture, faced with a record number of annual overdose deaths, we have an opportunity to turn the tide. Do we proceed “business-as-usual,” continuing to promote the same tired approaches, or do we instead follow the science by allocating resources to expand access to proven, life-saving treatments that work? Surging overdose deaths, coupled with consistently abysmal rates of medication adherence and treatment retention, make it clear that the time is now for contingency management for opioid use disorder (OUD).

Given the chronic, often relapsing course of severe OUD, effective treatment requires long-term management. Medication treatment with methadone or buprenorphine is a viable treatment option associated with a variety of positive outcomes, including most notably, reduced risk for all-cause and overdose mortality [4–7]. Poor adherence and premature discharge (i.e. early dropout) are widespread issues in office-based opioid treatment (OBOT) and opioid treatment program (OTP) settings [8,9]. Patients who are adherent to their medication, however, experience better outcomes [4,10,11]. In addition to FDA-approved medications, there are psychosocial interventions—such as contingency management—with demonstrated effectiveness for OUD [12]. Contingency management is a behavioural intervention wherein people are rewarded for achieving their treatment and recovery goals, often with monetary incentives. Seminal clinical trials from the 1990s provide strong evidence supporting contingency management and have since been systematically replicated nationally and internationally for over 30 years. Contingency management is an effective standalone or adjunctive intervention linked to longer periods of abstinence, longer treatment engagement, and greater improvements in social functioning among OUD populations [13–17]. Contingency management for OUD is associated with improved medication adherence and therapy attendance, as well as reduced misuse of opioids and other substances [12,18].

When combined with medication for OUD, contingency management demonstrates significant reductions in morbidity and mortality from OUD, considerable cost savings, and reduced hospitalizations and emergency department visits [19,20]. Contingency management is also associated with an overall medium-large effect size (*Cohen d* = 0.70) on abstinence from stimulants among patients receiving medication for OUD [12], which is salient in light of national trends showing accelerated overdoses involving stimulants and high rates of comorbid stimulant misuse with opioids [1,21,22]. The extant research base is clear: contingency management for OUD works, particularly as an adjunctive to medication treatment with methadone or buprenorphine.

## Barriers to implementation

Despite decades of research supporting the effectiveness of contingency management and generally positive beliefs held by front-line addiction treatment providers and patients alike [23,24], its application in real-world clinical settings is limited for a variety of reasons; many of which, as this paper will demonstrate, can be overcome. There are a number of obstacles described elsewhere regarding the low uptake of contingency management, including concerns about the durability of long-term effects and the potential for external reinforcement to impede intrinsic motivation to change [25–27]. The literature has largely found that contingency management does not have an adverse effect on readiness to change, and decades of support clearly indicate robust short-term benefits of contingency management with some evidence showing durable long-term improvements even after reinforcers are no longer delivered [25]. Barriers to implementation covered here will focus on the lack of innovation as well as the moral, philosophical, ethical, legal, and economic concerns often voiced by opponents of contingency management.

### Lack of innovation

A leading barrier to greater adoption of contingency management in clinical practice is presumably the relative lack of innovation. The origins of contingency management date back to the 1960s and operant conditioning principles, which was followed by a surge of rigorous, high-quality clinical trials in the 1990s. Traditional contingency management protocols (e.g. requirement for in-person appointments, use of a “prize bowl” filled with slips of paper) have since become rudimentary, outdated, and onerous in the current digital era, necessitating novel, technology-enabled solutions to facilitate widespread adoption. Other limitations include that many accepted contingency management procedures reward drug-free urinalysis screens exclusively, and there is only a low chance that the desired behaviour will actually be reinforced. In the commonly used probabilistic “prize-based” procedure, patients earn draws from a prize bowl containing slips of paper when the target behaviour is exhibited, but slips often have either no monetary value or a low-value prize. This raises the common complaint that contingency management is a “game of chance” due to the lack of immediate and consistent meaningful reinforcement that is required for lasting change.

There has been rapid progress on the innovation front in recent years with respect to emerging technologies leveraging contingency management in the treatment of OUD [20,28,29]. However, such advancements raise questions regarding potential disparities in access to technology-enabled, reward-based smartphone apps for some OUD populations, particularly individuals from low-income and racial/ethnic minority backgrounds. Although disadvantaged and underserved communities have traditionally been shown to have limited access to mobile health technologies and lower digital literacy, this gap is quickly narrowing [30]. Smartphones are becoming increasingly common with rates of ownership continuing to increase year-over-year for the U.S. general population [31]. Although Black and Hispanic adults are less likely than White adults to own a computer or have high-speed internet at home, national survey results indicate there are no racial/ethnic differences when it comes to smartphone ownership with 85% of Black, Hispanic, and White adults having a smartphone [31]. High rates of smartphone ownership have been documented for patients receiving outpatient addiction treatment [32] and even homeless populations [33]. Accumulating evidence suggests smartphone ownership, although certainly not universal, is no longer the barrier it once was. In light of the increasing penetration of smartphones users, and the fact that many patients already leverage technology in all facets of their lives, reward-based apps have the potential to bring contingency management into the hands of more people receiving treatment for OUD.

### Moral/philosophical/ethical concerns

A number of studies [34–36] have identified concerns voiced by treatment providers tasked with implementing contingency management (largely prize-based protocols) as well as patients, including the over-reliance on abstinence, fairness, perceived power imbalance, and how incentives will be spent. Opponents to contingency management may object on moral or philosophical grounds. The idea of rewarding someone to stop using drugs is counter to the oft-cited belief held by some treatment professionals—who are resistant to this key tenet of contingency management—that “patients have to want to get better.” A related criticism one may hear is that “patients will just use the money [from contingency management] to buy drugs,” rationalizing the withholding of evidence-based care on ethical grounds. This is consistent with prior work [36,37] demonstrating that one of the most commonly identified concerns about contingency management is the use to which any monetary incentives are put (i.e. “giving people ‘extra’ money at a vulnerable point in their treatment pathway may do more harm than good”). For those sharing these sentiments, I ask, do people with OUD not need money for basic human necessities such as groceries, rent, childcare, electricity, and other expenses? As a licenced clinical psychologist who has been involved in the design, implementation, delivery, and evaluation of clinical programming for a number of addiction treatment programs, I can confidently say that gainful employment—or more specifically, earning an income on the path to self-sufficiency—is strongly encouraged by treatment staff, particularly for patients early in their recovery. Whether it be linking patients to supported employment or vocational rehabilitation training programs, assistance with resume/cover letter writing and interview prep, facilitating job fairs, or providing other resources for those interested in or able to work, earning an income and money management are often addressed in the context of treatment and obtaining employment is viewed as a positive outcome [38]. I also find it important to highlight that the amount of monetary incentives that patients can expect to pocket by participating in a contingency management intervention is relatively minimal. Total earnings rarely exceed $100, on average, per month in most studied contingency management programs [39–41]. So why then is the opportunity for patients—many of whom may be unemployed when they begin treatment—to earn a few bucks each week for achieving their goals so controversial? This begs the question of whether ethical concerns about the perceived risk of harm in rewarding patients is about the money itself or perhaps has more to do with the person receiving the money. Paternalistic beliefs held by some addiction treatment providers, which I have witnessed firsthand and I am sure other treatment professionals can attest to, that they “know what is best” for the patient further contributes to the pervasive stigma that people who use drugs cannot be trusted with money, even small amounts contingent on positive treatment response.

Other opponents may argue that addiction treatment patients will try to "game" the system by simply doing whatever it takes to earn monetary rewards, thereby questioning patients’ true motivation “to get better.” When hearing this tired argument about people with addiction, I often find it helpful to consider patients diagnosed with a different chronic medical condition. Consider, for example, a patient with hypertension, diabetes, or obesity who earns $1 for each day they engage in 30 min of exercise, solely motivated by the modest financial incentive. Would this patient not still experience positive health benefits over time (lower blood pressure, weight loss, etc.) irrespective of their underlying motivation? Many health plans already offer incentives to their members who engage in various healthy lifestyle behaviours (e.g. counting steps, goal setting). What if we rewarded OUD patients with incentives for not only producing negative urinalysis drug screens, which is often the exclusive target behaviour of traditional contingency management protocols, but also engaging in recovery-oriented behaviours such as attending therapy sessions, taking their medication (buprenorphine, methadone), and participating in community-based mutual-help support groups? In fact, this broadened approach to contingency management rewards has been used to good effect in several studies [42,43], and the overemphasis on abstinence is viewed by treatment professionals as a leading barrier to more wide-scale adoption [34–36]. In light of the tragic death toll associated with the ongoing overdose crisis—and 275 Americans dying of an overdose every day [1]—I am less concerned with a patient's “why” regarding their motivation for engaging in treatment care and more focussed on keeping them alive so that they can achieve the benefits of recovery.

### Cost/legal constraints

Program administrators, policy-makers, and payers may understandably voice concerns about increased treatment costs associated with providing monetary incentives given that contingency management is often an “add-on” to usual care (i.e. adjunctive intervention). However, contingency management, when combined with medication treatment for OUD, has demonstrated the largest cost-savings relative to other evidence-based interventions for OUD, including medication alone [19]. A recent study examining the net impact of a digital therapeutic delivering contingency management *via* mobile app on medical costs due to hospital-based encounters and procedures among a sample of OUD patients treated with buprenorphine documented that the medical cost reduction in patients using the app relative to those receiving standard care offset the cost of the digital therapeutic itself, thereby resulting in a net cost-savings of $720 per patient [44].

The cost-saving benefits of reduced hospitalizations and emergency department visits for patients receiving contingency management, while certainly viewed as a positive outcome by treatment staff directly involved in the patient’s care, are often only realized by payers. Although many effective contingency management interventions are limited to around $100 a month in rewards [39–41], in the absence of more widespread reimbursement for contingency management services, most treatment programs are left to grapple with the decision to either absorb the costs associated with implementing contingency management or opt not to offer this effective, evidence-based service. Although there have been a number of recent strides in coverage for contingency management, including pilot programs in several states [45,46], many commercial and government insurers remain slow to cover contingency management.

Also of interest are legal concerns and whether the use of monetary incentives violates federal and state law because it could be considered unlawful to give patients money who are enrolled in federally- or state-funded health plans or programs. The federal anti-kickback statute provides for criminal penalties for providers who knowingly and wilfully offer, pay, solicit, or receive remuneration to induce or reward, among other things, the referral of business reimbursable under any of the federal health care programs (Medicare and Medicaid). When incentives exceed nominal monetary values, they can be considered kickbacks or inducements per federal and state laws intended to prevent fraud, waste, and abuse. Several leading professional organizations and advocacy groups (e.g. American Society of Addiction Medicine, American Psychiatric Association, American Academy of Addiction Psychiatry, Shatterproof) have called on the Office of Inspector General (OIG) to create a new safe harbour provision protecting the use of payments provided as part of contingency management for patients receiving treatment *via* federally-funded health plans or programs [47,48]. Establishing a safe harbour for contingency management, with common-sense guardrails in place to ensure its appropriate use, can help make contingency management more accessible.

The Office of Inspector General (OIG) published a final rule in December 2020 amending safe harbours to the federal anti-kickback statute [49]. Although the final rule did not expand the patient engagement and support safe harbour to include cash and cash-equivalent payments offered as part of contingency management interventions, the OIG clarified that this did not mean that all such cash or cash-equivalent payments are unlawful. Rather, they would be subject to case-by-case analysis under the federal anti-kickback statute, 42 U.S.C. § 1320a-7b(b), and the civil monetary penalty (CMP) law provision prohibiting inducements to beneficiaries (Beneficiary Inducements CMP), 42 U.S.C. § 1320a7a(a)(5). The OIG even went so far as to dispel the oft-stated assumption that the OIG bans incentives with a monetary value greater than $75, explicitly stating “there is no OIG-imposed $75 limitation on contingency management program incentives.” In accordance with their approach to evaluate contingency management programs on a case-by-case basis, the OIG posted an advisory legal opinion (OIG Advisory Opinion No. 22-04) in March 2022 approving the use of a digital contingency management program using smartphone and smart debit card technology, which could clear the way for wider use of similar programs in routine treatment settings.

The Biden-Harris administration has been transparent in its support for expanding access to evidence-based treatment, including contingency management [50]. The Office of National Drug Control Policy’s stated priorities include addressing policy barriers related to contingency management interventions, and exploring reimbursement for motivational incentives and digital treatment for addiction. The U.S. Surgeon General and several federal agencies and institutes, including the National Institute on Drug Abuse (NIDA), Substance Abuse and Mental Health Services Administration (SAMHSA), U.S. Food and Drug Administration (FDA), and the Department of Veterans Affairs, have all taken actions signalling acceptance of contingency management as an effective intervention. A number of states (e.g. California, Washington, Montana, West Virginia) are also pushing for change by actively pursuing legislation and appealing to federal regulators to make contingency management more widely available.

## Solutions to overcoming barriers

### Emerging technologies

Barriers to widespread adoption of contingency management in routine clinical practice, although pervasive, can be overcome. A number of innovative technologies, as described below, now allow for many aspects of contingency management to be fully or partially automated, thereby addressing common logistical barriers to implementation. Patient-facing mobile apps, combined with provider-facing dashboards, can facilitate tracking progress towards recovery goals and overall program-level management of the selected rewards system. Full automation is any solution not requiring action or verification by treatment staff before rewards can be delivered, whereas partial automation involves rapid delivery of rewards for certain recovery-oriented behaviours with other behaviours requiring verification by individual providers. Depending on the identified target behaviours, validation can be achieved *via* multiple easy and convenient methods. Supplementing patient or collateral self-report, smartphone video and GPS location capabilities, as well as external testing hardware have all been used to good effect to monitor and confirm medication adherence, abstinence, and appointment attendance [28,51–54]. Rewards contingent on abstinence can be delivered immediately using smartphone-linked remote breathalyzer/saliva drug testing or after verification by the provider following a negative urinalysis drug screen at routine in-person clinic visits. Similarly, rewards for attendance at scheduled outpatient appointments or community-based mutual-help support groups (e.g. Narcotics Anonymous) can be automatically delivered based on smartphone GPS location data in conjunction with start/stop timestamps, or delivered following manual verification by the provider after logging in to the dashboard to release earned rewards.

Automated delivery of monetary rewards can be achieved by linking a contingency management mobile app to a pre-paid debit card with the option to apply spending restrictions. “Smart” debit cards allow card administrators (e.g. treatment program staff) to toggle specified blocking capabilities on/off to prevent cash withdrawals or purchases at identified high-risk vendors (e.g. bars, liquor stores, casinos). Both providers as well as patients in OUD treatment settings overwhelmingly prefer the use of “smart” pre-paid debit cards relative to giving patients actual cash, and view spending restrictions as an appropriate safeguard, particularly early on in one’s recovery [24]. Clinically, providers and patients alike may find it useful to collaboratively identify high-risk vendors or spending categories to block with clearly outlined expectations for the eventual withdrawal of all spending restrictions over time based on patient preference and response to treatment. With technology-enabled contingency management, patients are also incentivized to provide clinically-meaningful outcomes data using the app, which can then be reviewed by the provider in real-time using the dashboard. The COVID-19 pandemic has forced OUD treatment programs to adapt workflows and embrace technology [55], especially with vulnerable populations, creating a unique opportunity to further incorporate innovative contingency management solutions into routine practice.

### Training

Education and training may be indicated to improve uptake of contingency management by addressing perceived skill deficits and competence needs of clinical staff [26,56], who often receive limited formal training in contingency management as part of their graduate coursework or licensure/certification requirements [57]. Traditional, largely didactic training approaches to scaling adoption of evidence-based interventions may be effective for enhancing staff knowledge, but are insufficient for sustained change in staff competence and skill, as well as patient outcomes [58]. Instead, preferred training strategies for contingency management scale-up in community treatment settings, as identified by opioid treatment program staff themselves, include the provision of a brief (half-day to 2 days) didactic training workshop supplemented with case examples and research data, along with experiential learning strategies such as role-paying [59]. Inclusion of case examples has been found to render evidence-based interventions more compelling and increase clinician interest in gaining training [60]. Several contingency management training and dissemination efforts have demonstrated robust, durable improvements in staff knowledge, delivery skill, and adoption readiness [61,62].

### Advocacy

Involvement in local, state, and federal advocacy efforts to make contingency management more mainstream is required to lessen treatment providers’ perceived exposure to risk in providing payments to patients as part of contingency management interventions. At the federal level, this involves continuing to push for a safe harbour provision protecting the use of motivational incentives for patients enrolled in federally-funded health plans or programs, and participating in public comment periods. A newly created safe harbour specifically covering contingency management would ensure that despite potentially implicating the federal anti-kickback statute, contingency management would not be treated as an offence under the statute. Addiction treatment professionals, including executives, clinical directors, and front-line clinicians, are encouraged to familiarize themselves with their local and state elected officials, as well as their own state’s statutes regulating contingency management and the use of motivational incentives for state-funded programs as a logical first step, and then taking action, if necessary. Following local, state, and federal legislators on social media (as well as leading addiction policy advocates and organizations) also allows the opportunity to keep abreast of the latest relevant policy-related issues impacting addiction treatment and recovery. Greater acceptance of contingency management involves not only fixing legislation and regulations preventing uptake, but recognizing stigma, shaping attitudes, and increasing awareness. In addition to legislative advocacy, clinical staff can become a local champion and change agent for contingency management at their treatment program or health care system by proposing in-service trainings and calling out stigma when present by patients, colleagues, or staff. They can use their voice to advocate for compassionate, common-sense approaches and expand access to evidence-based interventions like contingency management for people with OUD. Educating others, raising awareness, and holding policy-makers accountable are small but effective ways to have a measurable impact on uptake and adoption of contingency management.

### Academic-Industry collaboration

Academic-industry partnerships are critical to building, testing, and scaling technology-enabled, reward-based solutions for OUD. Such collaborations could substantially advance addiction treatment systems by jointly bringing intuitive, science-backed solutions to market, which serve to improve uptake of contingency management and increase the availability of treatment slots by leveraging the efforts of program staff, particularly in underserved areas, where staff shortages are common. Although there is great potential for technology-enabled solutions to further support the prevention, treatment, and recovery of OUD by addressing practical barriers to adoption, OUD mobile apps often lack empirical evidence from well-designed studies supporting their use [63]. As a number of mobile apps begin to emerge in the OUD treatment space, few meet basic quality standards. A recent survey of 619 opioid-related apps [64] identified 59 apps meeting criteria for quality assessment, but only a single app met basic quality standards, suggesting further work is warranted to fill this gap in technological solutions for OUD recovery management.

The U.S. digital health market is in the midst of considerable growth with total venture capital invested through mid-2021 at nearly $15 billion [65], surpassing last year’s record total funding in only six months. As funding continues to pour in for digital health startups, partnerships between the tech industry and academia are as important as ever to establish high-quality evidence of effectiveness from rigorous yet feasible pilot studies and publish findings in reputable peer-reviewed journals. Clinical researchers—and their distinctive skillset and depth of training in such areas as research design, methodology, statistical analysis, clinical interviews, focus groups, grant writing, and publishing—are uniquely positioned to bring a lot of added value to digital health companies in a scientific advisor/consultant role or by serving on boards. This is of paramount importance given that the overwhelming majority of digital health “unicorns” (i.e. companies valued at over $1 billion) lack any peer-reviewed papers supporting their products [66]. Researchers are adept at disseminating the findings from their research work in the form of peer-reviewed publications and presentations at national/international conferences.

The benefits of academic-industry partnerships flow both ways. Clinical researchers stand to gain just as much from the business know-how, technological expertise, marketing, and access to alternative and more streamlined lines of funding afforded to them by partnering with a digital health company. Moving beyond siloed academic work environments by forming strategic partnerships with industry provides researchers a front row seat to the practical application of their scientific findings, opens doors that may not have been possible otherwise, and is conducive to innovation. Recognizing the value of academic-industry collaboration, NIDA’s Small Business Technology Transfer (STTR) Program offers grant funding ranging from $150 K for Phase I up to $3 M for Phase II to foster technology transfer through cooperative R&D between academic researchers from non-profit universities and for-profit small business startups. A true bi-directional approach has the potential to shape and inform scientific research questions with an eye towards real-world challenges to implementation, ultimately improving contingency management solutions.

## Suggestions for future research

In order to further advance the extant knowledge base on contingency management in general and technology-enabled solutions in particular, there are a number of areas warranting further work. Efforts targeting barriers to more wide-scale acceptance and implementation of contingency management, including most notably the apparent lack of innovation, would benefit from further empirical studies identifying specific technology-related reasons cited by program administrators and front-line clinical staff for the low rates of adoption in real-world treatment settings. The overwhelmingly majority of contingency management research studies conducted to date come from the U.S., and even fewer studies have examined contingency management for OUD specifically outside of the U.S. [42,67]. A recent meta-analysis of studies where treatment providers targeted attendance behaviours, either in isolation or in combination with targeting abstinence, found that only one study was conducted outside of the U.S. [42]. Similarly, a 2016 review from the European Monitoring Centre for Drugs and Drug Addiction on the effectiveness of contingency management as an adjunctive to pharmacological interventions for substance use disorder found that of all high-quality studies included, only four were from countries besides the U.S. [67]. Surveys of mental health providers practicing in other countries reveal that, similar to the U.S., few are aware of contingency management and even fewer use the intervention in their routine practice [57]. Further research is therefore warranted to determine whether the positive findings observed for contingency management for OUD from U.S. samples generalize to other cultures.

It is also critical that contingency management researchers continue to expand on existing work to explore the feasibility and effectiveness of rewarding additional recovery-oriented behaviours beyond negative urinalysis drug screens. Although there is some evidence suggesting that rewarding attendance at appointments is equally effective to rewarding abstinence with respect to treatment engagement [42,43], further research is necessary to determine the additive effects of targeting both behaviours on treatment outcomes, as well as the potential value in rewarding medication adherence, completing evidence-based psychoeducational learning modules on identified topics of particular interest to OUD patients (naloxone, etc.), among other clinically-indicated yet understudied target behaviours. Finally, despite positive preliminary findings from several recent studies testing innovative technology-enabled solutions leveraging contingency management for OUD, additional rigorous, large-scale trials are needed, particularly with more racially and culturally diverse, underserved populations. Although there has been a proliferation of mobile apps for OUD in recent years [63,64], only a select few reward-based solutions have empirical evidence of preliminary effectiveness published in peer-reviewed journals [20,28], underscoring the need for greater collaboration between academic researchers and technology startups involved in the OUD treatment space.

## Conclusions

Contingency management must be used as a critical front-line treatment for OUD in conjunction with life-saving FDA-approved medications. Despite decades of rigorous clinical trials and robust meta-analyses showing considerable benefits for contingency management interventions, a number of barriers precluding widespread adoption persist. Fortunately, many barriers can be overcome by holding specialized staff trainings, actively calling out stigma, advocating locally and nationally, fostering academic-industry partnerships, and incorporating innovative technologies. Adoption of technology-enabled contingency management solutions, that reward more than simply negative drug screens to include additional recovery-oriented behaviours [42,43], is a requisite if enhanced rates of adherence and engagement, as well as quality of life improvements are to be achieved. Acceptance and uptake of reward-based interventions empirically shown to improve adherence to life-saving medications and retention in OUD treatment have the potential to curb the devastation currently felt by the ongoing opioid overdose crisis and ultimately save lives. Greater collaboration between digital health companies (with their access to qualified system engineers and potential to rapidly secure streamlined external investment) and clinical researchers (with their depth of expertise in relevant clinical treatment issues and unique training in research design and publishing) is necessary to build, validate, and scale-up intuitive, reward-based mobile apps for OUD. Academic researchers and industry professionals have unique, often complementary skillsets and resources that, when pooled together, have a real shot at turning the tide on the overdose crisis. Such collaboration will inevitably have a considerably greater impact at-scale on individuals and families affected by OUD than ever thought possible in their own siloed efforts. As America’s tragic overdose death toll continues to rise, we have also seen a number of technological advancements emerge in recent years, suggesting the time is now to rapidly accelerate the development and testing of innovative contingency management solutions.

## Data Availability

Data sharing is not applicable to this article as no new data were created or analysed in this study.
